# Prevalence of Dental Fear among 6-15 Years Old School Children

**DOI:** 10.31729/jnma.4791

**Published:** 2020-01-31

**Authors:** Sirjana Dahal, Ashish Shrestha, Tarakant Bhagat

**Affiliations:** 1 Department of Community and Public Health Dentistry, Kathmandu Medical College Teaching Hospital, Duwakot, Bhaktapur, Nepal; 2 Department of Public Health Dentistry, B.P. Koirala Institute of Health Sciences, Dharan, Nepal

**Keywords:** *children*, *dental fear*, *gender differences*, *Nepal*

## Abstract

**Introduction:**

Odontophobia or dental fear is a “unique phobia with special psychosomatic components that impact on the dental health of the odontophobia persons”. It is well documented that dental fear has a significant impact on dental care utilization behaviors. The objective of this study was to find out the level of dental fear among school children studying in government schools of Dharan, Nepal.

**Methods:**

A descriptive cross-sectional study was conducted from March to August 2017 among 215 school going children of Dharan of age group 6 to 15 years. Ethical approval was obtained. Children studying in six different government schools of Dharan were selected using two stage cluster sampling method. The Children's Fear Survey Schedule-Dental Subscale was used to measure dental fear among the study group. Data were entered in Microsoft Excel Sheet 2007 and analyzed in Statistical Package of Social Sciences 11.5.

**Results:**

This study showed that among the total study population, 96 (44.7%) had high fear, 62 (28.8%) had moderate fear and 57 (26.5%) had low dental fear. Among males, 29 (34.5%) had high fear whereas more than half of the female respondents had high fear.

**Conclusions:**

The present study showed that most of the school going children had high fear of dental treatment. So, school health programs should be planned to make the children familiar to dentistry and proper treatment modalities should be provided to make the child comfortable to seek dental care.

## INTRODUCTION

The technological advances have made dentistry less painful and less uncomfortable but problem of dental fear/anxiety still persists.^1^ Odontophobia or dental fear is a “unique phobia with special psychosomatic components that impact on the dental health of the odontophobic persons”.^2^ Fear of dentists has been ranked fourth among common fears.^3^ The prevalence of childhood dental fear varies from 6% to 52% depending upon how it is measured, age of the children and culture.^4^

Due to dental anxiety, patients often defer their dental treatment. This makes them suffer ultimately from inferior oral health as compared to non-anxious patients.^5^ Avoidance of dental care may lead to a vicious circle with the patient's dental problems mounting and leading to more unpleasant dental visits.^6^

This study was designed to find the level of dental fear among school children of Dharan, Nepal.

## METHODS

A community-based descriptive cross-sectional study was conducted from March to August 2017 among 215 school children of Dharan, Nepal. Students belonging to 6 to 15 years old age group were selected from six different Government schools of Dharan, Nepal, selected by two stage cluster sampling method. Ethical approval was obtained from Institutional Review Committee (IRC) BPKIHS, Dharan [Ref. no: IRC/632/015]. Written informed consent was obtained from the parents of children selected for study and assent was taken from the participants. Students who could properly read and write were included in the study. Differently abled and mentally challenged school children were excluded from the study.

With reference to the previous prevalence study on Dental Fear carried out by Bangalore, India^3^ (prevalence 46.8%), sample size was calculated by,

$$\begin{array}{ll} \text{n} & \text{= }{\text{Z}}^{\text{2}}\times \text{p(1}-\text{p)}/{\text{e}}^{2}\\ & \text{= 1}{\text{.96}}^{\text{2}}\times \text{0.468(1}-\text{0.468)}/\text{0}{\text{.07}}^{\text{2}}\\ & \text{= }195.12 \end{array}$$

where,

n = the minimum required sample size
Z = 1.96 at 95% confidence interval
p = prevalence of the facial injuries, 46.8%
q = 1-p
e = margin of error, 7%

Adding 10% of non-response, the total sample taken was 215.

The survey instrument including 15-item questionnaire of Children's Fear Survey Schedule-Dental Subscale (CFSS-DS) was used. Each question consists of five different scores ranging from one (not afraid at all) to five (very much afraid). Total scores ranged from 5 to 75. Score <31 was low fear, 31-39 was moderate fear, >39 was high fear.^7^

The questionnaire was translated into Nepali language by standard back translation method (forward and backward translation process) and used in this study. Face and content validity along with test-retest reliability of the questionnaire was done. The questionnaire was first pilot tested among 25 students of two government schools in Dharan, Nepal and was not included in the final sample. Minor changes were made to the questionnaire following the pilot phase to improve the clarity of questions.

The self-administered questionnaires were distributed to the students in their classrooms. They were explained about the purpose of the study and how to answer the questionnaire was also clarified. All the children present in the classes completed the questionnaire. The students were not allowed to confer or consult with each other. They completed responding to the questionnaire in 20-25 minutes.

After completion of data collection, data obtained were entered in Microsoft Excel Sheet version 2007 and analyzed using the Statistical Package for Social Sciences version 11.5. Descriptive statistics, including the mean and standard deviations were computed for all the items of dental fear. Internal consistency of the questionnaire was evaluated using Cronbach's alpha and intra-class correlation coefficient was used for determining reliability. Frequency distribution in level of dental fear among the study population was calculated.

## RESULTS

**Figure 1.**
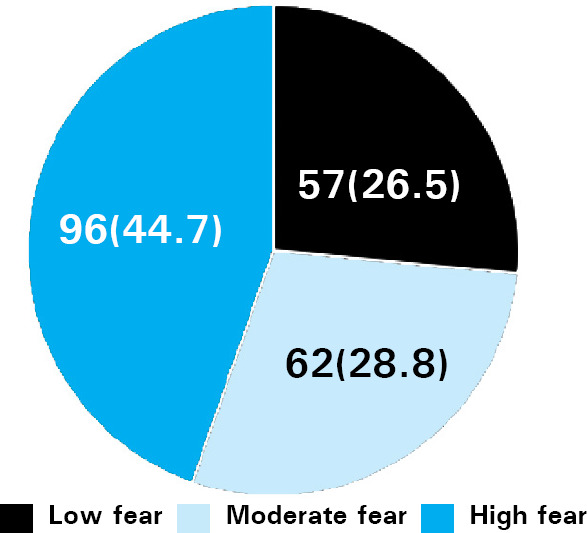
Prevalence of dental fear.

This study showed that most of the school children 96 (45.1%) had high fear (Figure 1).

Two hundred and fifteen children participated in the study among which 84 (39.1%) were males and 131 (60.9%) were females. The response rate was 100%. The mean age of the school children was 10.17±1.623 years.

**Figure 2.**
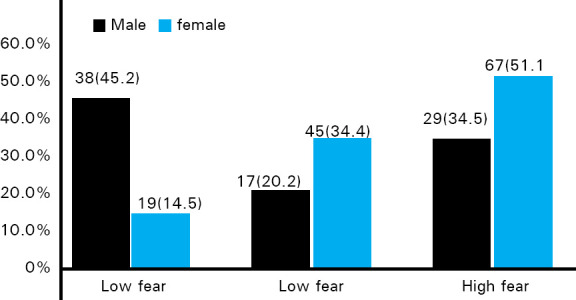
Level of dental fear among males and females.

Females 67 (51.1%) had significantly high fear than males 29 (34.5%) (Figure 2).

Generally, girls tended to have higher mean values than boys in most items, rating them more fearful than boys (Table 1).

**Table 1:** Individual items scores of CFSS-DS scales.

S. N	Source of fear	Overall Mean±SD	Mean±SD (Males)	Mean±SD (Females)
1	Dentist	2.43±1.162	2.24±1.037	2.56±1.223
2	Doctors	2.71±1.172	2.44±1.144	2.89±1.161
3	Injections	2.70±1.205	2.33±1.186	2.93±1.163
4	Having somebody examine your mouth	2.28±1.191	2±0.944	2.47±1.297
5	Having to open your mouth	2.31±1.172	2.07±1.106	2.47±1.192
6	Having a stranger touch you	2.70±1.236	2.55±1.155	2.80±1.28
7	Having somebody to look at you	2.28±1.260	2.15±1.116	2.37±1.343
8	The dentist drilling	2.95±1.163	2.70±1.128	3.11±1.161
9	The sight of dentist drilling	2.78±1.129	2.43±0.098	3.01±1.180
10	The noise of dentist drilling	2.87±1.105	2.67±1.057	2.99±1.120
11	Having somebody put instrument in your mouth	2.98±1.236	2.87±1.149	3.05±1.288
12	Choking	2.30±1.154	2.04±0.936	2.47±1.236
13	Having to go to hospital	2.44±1.213	2.12±1.046	2.65±1.271
14	People in white uniform	2.43±1.197	2.35±1.114	2.48±1.249
15	Having the nurse clean your mouth	2.45±1.186	2.32±1.110	2.53±1.230

**Figure 3.**
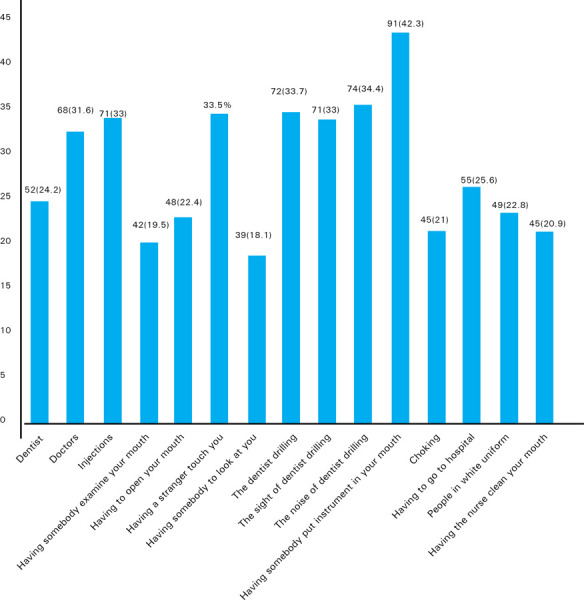
Each item eliciting the highest fear in children.

The percentage of fearful children (combining the responses of pretty much afraid and very much afraid) for each item was calculated (Figure 3). Ninety-one (42.3%) out of 215 were anxious about having instruments placed in their mouth and 74 (34.4%) feared on noise of dentist drilling. The least of children surveyed 39 (18.1%) were found fearful on having somebody to look at them.

## DISCUSSION

Dental fear refers to a normal protective reaction to a real threat, the feeling of which disappears when the threat is no longer present. Dental anxiety is more of a subjective state of feeling which is often associated with a feeling of danger.^1^ Despite the advancement in techniques, technologies and materials as well as improvement in public awareness of oral health, dental anxiety still persists as a major problem worldwide and remains a significant challenge in improving dental care.^8^

This study represents an attempt to find the prevalence of dental fear among 6 to 15 years old school children of Dharan. The mean score of CFSS-DS in this study was 38.60±9.39 which is similar to that of studies done in Banglore, India and Singapore where the mean score was 30.6±10.8 and 37.0±8.89, respectively.^3,9^

Most of the participants 159 (74%) in the study had moderate to high dental fear. This may be due to the fact that most of the children do not get exposed to dentistry at an early age and they are not familiar with the dental environment.

Females showed higher fear than males which is consistent with various other studies done in regard to dental fear.^1,4,10^ This may be due to higher chance of having neuroticism in females which is governed by characteristics of being anxious, angry and jealous or because males tend to hide their fears due to their conventional gender role.^5^ However, some studies showed no difference in fear among males and females.^11,12,13^ Findings with regard to effect of gender on dental anxiety may be varied in studies done in different places due to culture differences.^14^

The school children who participated in this study were mostly afraid of 'having somebody put instruments in their mouth. This could be due to the child's fear of pain which he/she anticipates during dental treatment. Other potent triggers for dental fear in this study population were particularly the sight, sound and sense of vibration of rotary drills as well as the sight and sensation of local anesthetic injections. However, in most of the studies, children showed highest level of fear on 'injections'.^3,9,13^ Only few children in this study showed high fear on 'having somebody look at them' and 'on having somebody examine their mouth' which was similar to the study done in Singapore and Bengaluru, India.^9,13^

Dental fear/anxiety in children is a very serious problem that can have negative impact on oral health. So, it is very essential to detect the cause of fear at an early stage and find a solution so as to make the child comfortable to dental environment. Currently, there are several management techniques such as pretreatment anxiety questionnaires, flooding/implosion, cognitive behavioral therapy, relaxation therapy, computer-assisted relaxation learning, hypnotherapy and pharmacological therapy coming up in order to overcome dental fear and anxiety.^15^

The present cross-sectional study has some limitations. The study has been conducted in a six different government schools of Dharan, Nepal. Hence, studies on larger populations are needed to reveal prevalence and determinants of dental fear among children in a larger scale. Since, self-administered questionnaires were used, information and response bias could not be managed.

## CONCLUSIONS

The study showed that most of school children had high dental fear as measured according to CFSS-DS scale and was predominant in females. Oral instrumentation and drilling were the most common causes of fear. The results of the study suggest that regular oral health awareness programs at schools and proper treatment modalities with behavior modification techniques should be provided to make the child comfortable to seek dental care.
